# In Utero Exposure to Maternal SARS-CoV-2 Infection Is Associated With Higher Left Ventricular Mass in Toddlers

**DOI:** 10.1093/ofid/ofae305

**Published:** 2024-05-31

**Authors:** Mollie W Ockene, Duraisamy Balaguru, Ingrid L Ma, Samuel C Russo, Allison K Arpante, Alexandra Clifford, Olyvia J Jasset, Joon H Kim, Mabel Toribio, Takara L Stanley, Lydia L Shook, Andrea G Edlow, Lindsay T Fourman

**Affiliations:** Metabolism Unit, Massachusetts General Hospital, Boston, Massachusetts, USA; Pediatric Cardiology, Massachusetts General Hospital, Boston, Massachusetts, USA; Metabolism Unit, Massachusetts General Hospital, Boston, Massachusetts, USA; Metabolism Unit, Massachusetts General Hospital, Boston, Massachusetts, USA; Metabolism Unit, Massachusetts General Hospital, Boston, Massachusetts, USA; Metabolism Unit, Massachusetts General Hospital, Boston, Massachusetts, USA; Vincent Center for Reproductive Biology, Massachusetts General Hospital, Boston, Massachusetts, USA; Vincent Center for Reproductive Biology, Massachusetts General Hospital, Boston, Massachusetts, USA; Metabolism Unit, Massachusetts General Hospital, Boston, Massachusetts, USA; Metabolism Unit, Massachusetts General Hospital, Boston, Massachusetts, USA; Vincent Center for Reproductive Biology, Massachusetts General Hospital, Boston, Massachusetts, USA; Department of Obstetrics and Gynecology, Maternal-Fetal Medicine Division, Massachusetts General Hospital and Harvard Medical School, Boston, Massachusetts, USA; Vincent Center for Reproductive Biology, Massachusetts General Hospital, Boston, Massachusetts, USA; Department of Obstetrics and Gynecology, Maternal-Fetal Medicine Division, Massachusetts General Hospital and Harvard Medical School, Boston, Massachusetts, USA; Metabolism Unit, Massachusetts General Hospital, Boston, Massachusetts, USA

**Keywords:** cardiometabolic disease, COVID-19, developmental origins of disease, in utero, left ventricular mass

## Abstract

The intrauterine environment plays a critical role in shaping chronic disease risk over the life course. We prospectively evaluated cardiometabolic outcomes in toddlers born to mothers with versus without prenatal severe acute respiratory syndrome coronavirus 2 infection. Children with in utero severe acute respiratory syndrome coronavirus 2 exposure had higher left ventricular mass in association with altered maternal immunologic indices.

## Graphical Abstract

**Graphical Abstract:**
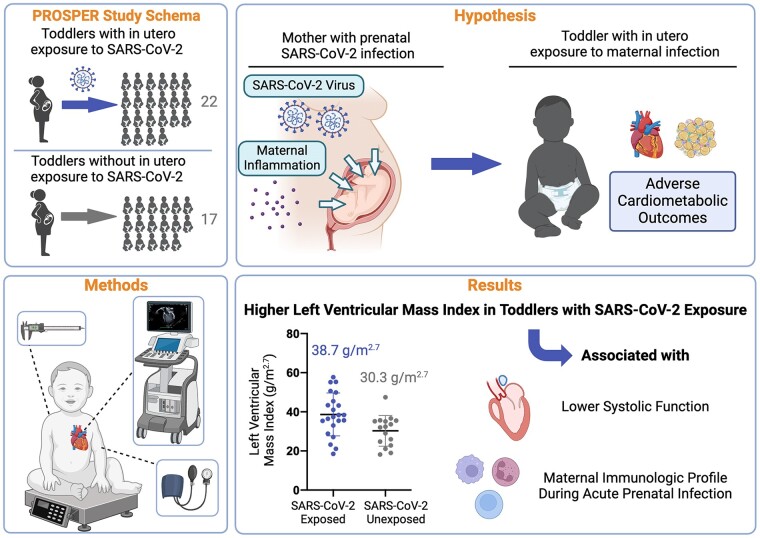

Since the initial outbreak of the coronavirus disease 2019 (COVID-19) pandemic, a novel population of children with in utero exposure to maternal severe acute respiratory syndrome coronavirus 2 (SARS-CoV-2) infection has emerged [1]. Because the intrauterine environment plays a critical role in shaping chronic disease risk over the life course, it is imperative to evaluate health outcomes among this expanding group [2]. In a retrospective study, we found that individuals with in utero exposure to maternal SARS-CoV-2 infection exhibited a differential growth pattern in the first year of life, characterized by lower birth weight and accelerated weight gain [3]. In another health records-based cohort, we similarly identified higher rates of neurodevelopmental [4] and cardiometabolic disorders [5] in infants and toddlers born to mothers with prenatal SARS-CoV-2 infection versus unexposed controls. These studies relied on health records to characterize the first 12 to 18 months of life. In the current pilot study, In Utero Exposure to SARS-CoV-2 and Cardiovascular and Metabolic Endpoints in Early Life, we performed a prospective evaluation of cardiometabolic outcomes among children with versus without in utero exposure to maternal SARS-CoV-2 infection. We hypothesized that toddlers born to mothers with prenatal SARS-CoV-2 infection would exhibit an altered cardiometabolic phenotype in early life that may presage adverse health sequelae in the long term.

## METHODS

### Study Participants

Mothers with and without prenatal SARS-CoV-2 infection and their toddlers (12–24 months) who previously participated in the Mass General Brigham COVID-19 Pregnancy Biorepository [1] were invited to participate in this pilot study. The following exclusion criteria, assessed at study screen, were additionally applied: (1) child with history of birth from a multiple gestation pregnancy, SARS-CoV-2 infection or vaccination, congenital heart disease, or kidney disease; (2) mother with history of SARS-CoV-2 vaccination before delivery, chronic infection (eg, HIV), diabetes mellitus, chronic hypertension, or systemic autoimmune disease; and (3) significant illness in mother or child representing a contraindication to participation. Written informed consent was obtained from mothers between June 2021 and September 2022. The Mass General Brigham institutional review board approved this study.

### Procedures

At a single study visit, weight, length, skinfold thicknesses at triceps and subscapular sites, and blood pressure were measured in toddlers and normalized for age and sex to World Health Organization or American Academy of Pediatrics standards [6–8]. Echocardiography also was performed (EPIQ CVx, Philips, Amsterdam, Netherlands) and read by a single cardiologist (D.B.) blinded to study group (Supplementary Methods). Left ventricular (LV) mass was calculated using the 5/6 × area × length method [9–11] and indexed to length raised to an exponential power of 2.7 [8, 12], which is denoted here as LV mass index (LVMI). Prenatal history and laboratory parameters were obtained from mothers using standardized questions and/or manual review of medical records. Birth weight *z*-scores were calculated using Fenton growth curves, which account for gestational age and sex [13]. Mothers completed the MacArthur Scale of Subjective Social Status [14, 15], the U.S. Household Food Security Survey Module [16], and the Holmes-Rahe Stress Inventory Scale [17].

### Statistical Analysis

Characteristics were compared between groups using 2-sample *t*-test (continuous variables, normally distributed), Wilcoxon rank-sum test (continuous variables, not normally distributed), and chi-square test (categorical variables). Multivariable linear regression models were used to assess for differences between groups upon adjusting for key sociodemographic and biologic parameters. Associations within the SARS-CoV-2 exposure group were evaluated using Spearman's rank-order correlation. Variables were expressed as mean ± standard deviation (normally distributed) or median [interquartile range] (not normally distributed). *P* < .05 was used to designate statistical significance. Correction for multiple comparisons was not performed because of the exploratory nature of this pilot study. Analyses were performed using JMP 16.2.0 (SAS Institute Inc., Cary, North Carolina, USA).

## RESULTS

### Maternal and Infant Characteristics

A total of 22 mother-toddler dyads with maternal prenatal SARS-CoV-2 infection and 17 mother-toddler dyads without prenatal SARS-CoV-2 infection were enrolled (Supplementary Figure 1; Supplementary Table 1). Higher rates of public insurance (59% vs 18%) and pregravid obesity (36% vs 12%) and a lower rate of college degree (45% vs 88%) were observed among mothers with versus without infection. Rates of preeclampsia and gestational hypertension were comparable between groups. Toddlers in both groups were of similar age (17 ± 2 months, range 14–20 months) and sex (56% male). Hispanic/Latinx ethnicity was more prevalent among babies born to mothers with versus without infection (73% vs 35%). Mothers with prenatal SARS-CoV-2 infection were diagnosed between March and December 2020 by reverse transcription-polymerase chain reaction. A total of 45% of mothers had moderate disease per National Institutes of Health criteria [18], with all infections occurring during the second or third trimesters.

### Cardiometabolic Outcomes by In Utero Exposure Status to Maternal SARS-CoV-2 Infection

**Figure 1.**
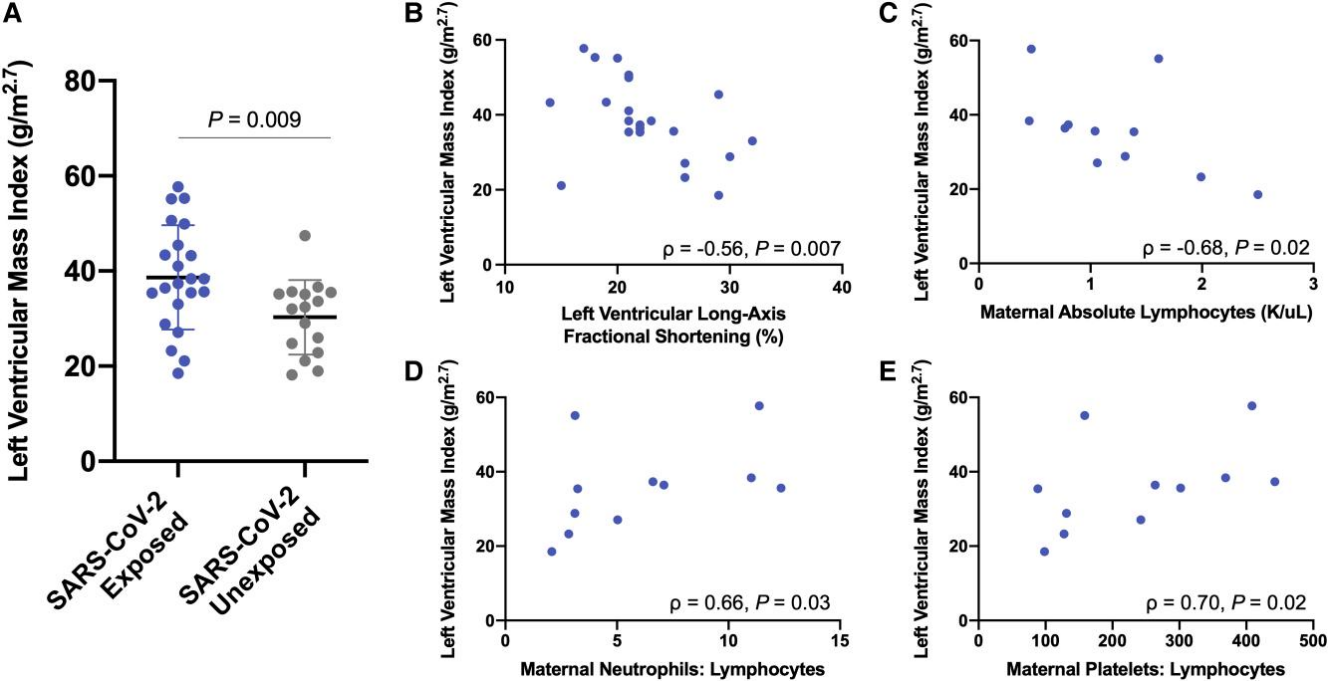
Association of in utero exposure to maternal SARS-CoV-2 infection with left ventricular mass index in toddlers. *A*, LVMI was higher in toddlers born to mothers with versus without prenatal SARS-CoV-2 infection using a 2-sample *t*-test (38.7 ± 11.0 vs 30.3 ± 7.8 g/m^2.7^, *P* = .009). Line and error bars denote mean and standard deviation, respectively. *B*, Among children with in utero SARS-CoV-2 exposure, LVMI correlated inversely with long-axis fractional shortening (ρ = −0.56, *P* = .007) as a measure of systolic function. *C-E*, Within the SARS-CoV-2 exposure group, LVMI in toddlers correlated inversely with maternal absolute lymphocyte count (ρ = −0.68, *P* = .02) and directly with maternal neutrophil: lymphocyte count (ρ = 0.66, *P* = .03) and platelet: lymphocyte count (ρ = 0.70, *P* = .02) during acute infection. Dot plots are shown with associations assessed using Spearman correlation.

We compared anthropometric and cardiovascular outcomes between toddlers with versus without in utero exposure to maternal SARS-CoV-2 infection (Table 1). Weight, length, body mass index, skinfold thickness, and blood pressure *z*-scores did not differ between groups. LVMI was higher in toddlers born to mothers with prenatal SARS-CoV-2 infection versus controls (38.7 ± 11.0 vs 30.3 ± 7.8 g/m^2.7^, *P* = .009) (Figure 1*A*). Nonetheless, there was no significant difference in frequency of LV hypertrophy (defined as LVMI > 51 g/m^2.7^ [8]) between the exposed and unexposed groups (14% vs 0%, *P* = .12). Toddlers with in utero SARS-CoV-2 exposure had higher LV mass: length ratio (4.6 ± 1.3 vs 3.8 ± 1.1 g/cm, *P* = .04) and tended to have higher LV mass: volume ratio (0.8 ± 0.2 vs 0.7 ± 0.3 g/mL, *P* = .12), consistent with concentric rather than eccentric remodeling. Although there was no difference between groups in LV function, LVMI was found to inversely correlate with long-axis fractional shortening (ρ = −0.56, *P* = .007) as a measure of systolic function within the SARS-CoV-2 exposure group (Figure 1*B*). No other significant differences between groups in cardiac structure or function were observed.

**Table 1.** Cardiometabolic Outcomes in Toddlers by In Utero SARS-CoV-2 Exposure Status

	Toddler With In Utero SARS-CoV-2 Exposure (n = 22)	Toddlers Without In Utero SARS-CoV-2 Exposure (n = 17)	*P* Value
Anthropometrics^a^			
Weight *z*-score	0.46 ± 0.85	0.67 ± 0.80	.43
Length *z*-score	−0.10 ± 0.89	0.38 ± 0.68	.06
BMI *z*-score	0.72 ± 0.93	0.63 ± 0.84	.74
Triceps skinfold *z*-score	1.18 ± 1.31	1.64 ± 1.17	.26
Subscapular skinfold *z*-score	0.98 ± 1.73	0.60 ± 1.53	.47
Echocardiography			
*Cardiac morphometry*			
LV end-diastolic volume, mL	26.3 ± 4.2	26.8 ± 6.3	.80
2D LV end-diastolic free wall thickness, cm	0.3 ± 0.1	0.3 ± 0.1	.95
LV mass index, g/m^2.7^	38.7 ± 11.0	30.3 ± 7.8	.**009**
LV mass: volume ratio, g/mL	0.8 ± 0.2	0.7 ± 0.3	.12
LV mass:length ratio, g/cm	4.6 ± 1.3	3.8 ± 1.1	.**04**
*Systolic function*			
2D LV long-axis fractional shortening, %	22 ± 5	23 ± 5	.55
LV ejection fraction, %	65 ± 5	65 ± 8	.93
*Diastolic function*			
Mitral E/A ratio	1.4 [1.3–1.7]	1.4 [1.3–1.5]	.98
*Vascular function* ^b^			
Systolic blood pressure *z*-score	1.08 ± 0.91	0.91 ± 0.75	.55
Diastolic blood pressure *z*-score	1.75 [1.29–2.05]	1.65 [1.17–2.33]	.82

Values are expressed as mean ± standard deviation or median [interquartile range]. Bold text denotes *P* < .05 as the predefined threshold for statistical significance. Sample sizes for individual parameters were as follows (toddlers with vs without in utero SARS-CoV-2 exposure): weight, length, BMI, and subscapular skinfold *z*-scores (22, 17); triceps skinfold *z*-score (22, 16); cardiac morphometry (22, 16); systolic function (22, 16); diastolic function (16, 11); vascular function (22, 16).

Abbreviations: 2D, 2-dimensional; BMI, body mass index; LV, left ventricle; SARS-CoV-2, severe acute respiratory syndrome coronavirus 2.

^a^Anthropometric *z*-scores were calculated using the World Health Organization standard curves, which account for age and sex.

^b^Blood pressure *z*-scores were calculated using the American Academy of Pediatrics standard curves, which account for age, sex, and height.

### Association of In Utero SARS-CoV-2 Exposure With Higher Left Ventricular Mass in Multivariable Models

In utero exposure to maternal SARS-CoV-2 infection remained associated with higher LVMI in a model adjusting for child age, sex, ethnicity, and history of breastfeeding as well as maternal insurance, education, and pregravid obesity (effect size: 10.5 g/m^2.7^; 95% confidence interval [CI], 2.6–18.4 g/m^2.7^, *P* = .01). This difference remained significant in a model additionally adjusting for preterm birth and birth weight *z*-score (effect size: 11.4 g/m^2.7^; 95% CI, 2.9–19.8 g/m^2.7^, *P* = .01). In a sensitivity analysis, LV mass indexed to body surface area was higher among toddlers born to mothers with versus without prenatal SARS-CoV-2 infection in a fully adjusted model (effect size: 11.1 g/m^2^, 95% CI, 2.4–19.9 g/m^2^, *P* = .01) (Supplementary Table 2).

### Relationships of Maternal SARS-CoV-2 Infection Characteristics With LV Mass

We performed an exploratory analysis to relate immunologic parameters in mothers with prenatal SARS-CoV-2 infection to LVMI in toddlers among the subset for whom complete blood count with differential was obtained clinically during active infection (n = 11). LVMI in toddlers was found to correlate inversely with maternal absolute lymphocyte count (ρ = −0.68, *P* = .02) and directly with maternal neutrophil: lymphocyte count (ρ = 0.66, *P* = .03) and platelet: lymphocyte count (ρ = 0.70, *P* = .02) (Figure 1*C*–*E*). There was no significant association between monocyte or neutrophil count and LVMI, nor between clinical infection severity [18] and LVMI.

## DISCUSSION

During fetal development, the cardiovascular system is exquisitely sensitive to intrauterine environmental cues. Although adaptive in the short term, changes in cardiac structure and function set in motion by the intrauterine environment may be seen postnatally and increase susceptibility to disease later in life [19, 20]. In the current study, we found that, among the cardiac parameters measured, mean LVMI was approximately 30% higher in toddlers with versus without in utero exposure to maternal SARS-CoV-2 infection. Furthermore, within the exposed group, higher LVMI was associated with lower long-axis fractional shortening, a measure of systolic function for which lower values denote worse function. Our findings in the setting of SARS-CoV-2 are consistent with prior reports of LV remodeling among offspring in other contexts, including maternal obesity [21, 22] and diabetes [23, 24] as well as preterm birth [25, 26] and intrauterine growth restriction [27, 28]. The persistence of an association between in utero SARS-CoV-2 exposure and LVMI, even on adjusting for or excluding these conditions, suggests that the effect of maternal SARS-CoV-2 infection on fetal cardiovascular programming may be independent of established early-life risk factors. Of note, we found no difference in blood pressure among toddlers between groups as a common cause of LV remodeling [29]. Although higher LV mass is a harbinger of adverse cardiovascular outcomes in adults [8, 30, 31], long-term clinical implications for this patient population require further evaluation over time.

Maternal inflammation has been posited as a central mechanism in the fetal programming of cardiometabolic disease [2, 32]. In this regard, sheep exposed to maternal inflammation in late gestation exhibited hearts with impaired structure and function and higher collagen deposition [33, 34]. In the current study, we analyzed relationships of LVMI with maternal immunologic indices derived from a complete blood count. This laboratory assessment was commonly available during acute infection as part of routine clinical care. We found that higher LVMI in toddlers was associated with immunologic alterations in mothers during prenatal SARS-CoV-2 infection, characterized by lower lymphocyte count and higher platelet:lymphocyte and neutrophil:lymphocyte ratios. The latter 2 parameters have been regarded as markers of disease severity and systemic inflammation in the setting of COVID-19 [35–37]. Future studies should delineate biologic pathways whereby inflammation in mothers with prenatal infection may contribute to perturbed cardiovascular structure and function in offspring.

To our knowledge, this study constitutes the first prospective investigation of cardiometabolic sequelae among children with in utero exposure to maternal SARS-CoV-2 infection. As a strength of this analysis, we recruited participants from a perinatal biorepository such that the SARS-CoV-2 status of each mother was assured and all mothers were pregnant during the pandemic to control for the impact of pandemic-associated stress. Furthermore, strict exclusion criteria were applied to minimize the heterogeneity of our sample. As a limitation of this study, the sample size was relatively small and thus the power to detect associations was limited. Likewise, there is risk of type I error in this hypothesis-generating analysis given use of multiple comparisons. Sociodemographic differences also were present between groups consistent with population-level trends regarding the COVID-19 pandemic [38] but were rigorously captured and adjusted for in multivariable models.

Our findings, although preliminary, suggest that maternal SARS-CoV-2 infection during pregnancy may pose risk to the long-term cardiovascular health of offspring. Large-scale longitudinal studies are needed to validate the cardiac abnormalities that we observed and to clarify their clinical significance over time. Insights gained in the SARS-CoV-2 context may more broadly illuminate the role of maternal infectious and inflammatory states in the fetal programming of cardiometabolic disease.

## Supplementary Data

Supplementary materials are available at the *Journal of The Pediatric Infectious Diseases Society* online (http://jpids.oxfordjournals.org). Supplementary materials consist of data provided by the author that are published to benefit the reader. The posted materials are not copyedited. The contents of all supplementary data are the sole responsibility of the authors. Questions or messages regarding errors should be addressed to the author.

## Supplementary Material

ofae305_Supplementary_Data

## Notes

***Acknowledgments.*** We thank the research volunteers whose generous commitment of time and effort made this study possible. We also thank the staff of the MGH Translational and Clinical Research Center and the Pediatric Cardiology Program at Mass General for Children for their dedication to patient care. M.W.O. contributed to investigation, data curation, data analysis, results interpretation, and manuscript preparation. D.B. contributed to investigation, results interpretation, and manuscript preparation. I.L.M. contributed to investigation, data curation, and manuscript preparation. S.C.R. contributed to data analysis, results interpretation, and manuscript preparation. A.K.A., A.C., O.J.J., J.H.K., M.T., T.L.S., and L.L.S. contributed to results interpretation and manuscript preparation. A.G.E. contributed to study conceptualization and design, data analysis, results interpretation, and manuscript preparation. L.T.F. contributed to study conceptualization and design, investigation, data curation, data analysis, results interpretation, and manuscript preparation. The graphical abstract for this manuscript was created with BioRender.com. Datasets generated during this study are not publicly available but are available from the corresponding author on reasonable request.

***Patient consent statement.*** Ethical approval was obtained from the Mass General Brigham institutional review board. This study (2021P001107) constituted a separate protocol from the parent pregnancy biorepository (2020P000804). Written informed consent was obtained from mothers on behalf of themselves and their children before participation in this study.

***Financial support*.** This work was supported by the National Institutes of Health [K23HL147799-01 to M.T.; 1K12HD103096 to L.L.S.; 1R01HD100022-01, 3R01HD100022-02S2 to A.G.E.; Harvard Nutrition Obesity Research Center at Harvard Pilot and Feasibility grants to A.G.E and L.T.F. (parent grant P30DK040561); Boston Area Diabetes Endocrinology Research Centers Pilot and Feasibility grant to A.G.E. (parent grant 5P30DK057521); K23HD100266 to L.T.F.; and 1UL1TR002541-01 to the Harvard Clinical and Translational Science Center]; American Heart Association-Harold Amos Medical Research Faculty Development Program by the Robert Wood Johnson Foundation [to M.T.]; and Simons Foundation [SFARI Award Grant 870754 to A.G.E.].

***Potential conflicts of interest*.** T.L.S. serves as a consultant to RosVivo Therapeutics and has grant funding to her institution from Pfizer, Inc. outside this work. A.G.E. is a consultant to Mirvie, Inc. and receives research funding from Merck Pharmaceuticals outside this work. L.T.F. serves as a consultant to Amryt Pharmaceuticals and Theratechnologies and receives grant funding to her institution from Amryt Pharmaceuticals outside of this work. All other authors report no potential conflicts.
