# Supplementary material for: In Utero Exposure to Maternal SARS-CoV-2 Infection Is Associated With Higher Left Ventricular Mass in Toddlers
Source: Open Forum Infect Dis. 2024 May 31;11(6):ofae305. doi: 10.1093/ofid/ofae305 (PMC11204912; doi:10.1093/ofid/ofae305)
Supplement: ofae305_Supplementary_Data [file ofae305_supplementary_data.zip › Supplemental Methods.pdf]

Supplemental Methods

Since performing echocardiography in toddlers may be challenging, we developed a protocol to efficiently ascertain a basic set of cardiac parameters for the purposes of this study. Echocardiography was performed on an EPIQ CVx machine (Philips, Amsterdam, Netherlands) using custom settings for children. Two-dimensional images in apical four-chamber and parasternal short-axis views were used to ascertain morphometric parameters. Left ventricular (LV) lengths were measured in apical four-chamber view from freeze frame images during end-systole and end-diastole and used to calculate LV long-axis fractional shortening. LV diameter and thicknesses of the interventricular septum and LV free (posterior) wall also were measured from end-diastolic freeze frame images in parasternal short-axis view. Freeze frame images during end-diastole and end-systole in parasternal short-axis view were further used to determine LV cross-sectional areas by tracing the endocardial and epicardial borders. LV diastolic and systolic volumes and LV mass were calculated using the 5/6 × area × length method [1-3]. LV volumes, in turn, were used to derive LV ejection fraction. Mitral valve inflow Doppler was obtained in apical four-chamber view using pulse-wave Doppler.

1. Myerson SG, Montgomery HE, World MJ, Pennell DJ. Left ventricular mass: reliability of M-mode and 2-dimensional echocardiographic formulas. Hypertension **2002**; 40(5): 673-8.
2. Lai WW, Geva T, Shirali GS, et al. Guidelines and standards for performance of a pediatric echocardiogram: a report from the Task Force of the Pediatric Council of the American Society of Echocardiography. J Am Soc Echocardiogr **2006**; 19(12): 1413-30.
3. Silverman NH, Schiller NB. Cross sectional echocardiographic assessment of cardiac chamber size and ejection fraction in children. Ultrasound Med Biol **1984**; 10(6): 757-69.
