# Supplementary material for: In Utero Exposure to Maternal SARS-CoV-2 Infection Is Associated With Higher Left Ventricular Mass in Toddlers
Source: Open Forum Infect Dis. 2024 May 31;11(6):ofae305. doi: 10.1093/ofid/ofae305 (PMC11204912; doi:10.1093/ofid/ofae305)
Supplement: ofae305_Supplementary_Data [file ofae305_supplementary_data.zip › Supplemental Table 1.pdf]

Supplemental Table 1. Maternal and Toddler Characteristics by In Utero SARS-CoV-2 Exposure Status

	Toddlers with In Utero SARS-CoV-2 Exposure (n = 22)	Toddlers without In Utero SARS-CoV-2 Exposure (n = 17)
**Maternal Characteristics**		
Age at delivery, years	33 ± 5	35 ± 5
Race		
White, n (%)	12 (55)	14 (82)
Black/African American, n (%)	1 (5)	0 (0)
Unknown or other, n (%)	9 (41)	3 (18)
Hispanic or Latina, n (%)	15 (68)	5 (29)
Public insurance, n (%)	13 (59)	3 (18)
College degree earned, n (%)	10 (45)	15 (88)
Perceived community social status[1]		
Lower tier	4 (18)	0 (0)
Middle tier	6 (27)	5 (29)
Upper tier	12 (55)	12 (71)
Household food insecurity, n (%)[2]	3 (14)	1 (6)
Depression, n (%)	6 (27)	4 (24)
Holmes-Rahe Stress Inventory Scale	164 ± 117	137 ± 76
**Maternal Obstetric History**		
Gravida	3 [2 - 4]	2 [1 - 3]
Parity	2 [1 - 3]	2 [1 - 2]
Pregravid obesity, n (%)	8 (36)	2 (12)
Preeclampsia, n (%)	3 (14)	1 (6)
Gestational hypertension, n (%)	3 (14)	0 (0)
Tobacco use in pregnancy, n (%)	0 (0)	0 (0)
Preterm, n (%)	5 (23)	1 (6)
Cesarean section, n (%)	10 (45)	12 (71)
Severity of SARS-CoV-2 infection		
Asymptomatic, n (%)	5 (23)	-
Mild, n (%)	4 (18)	-
Moderate, n (%)	10 (45)	-
Severe, n (%)	3 (14)	-
Trimester of SARS-CoV-2 infection		
First trimester, n (%)	0 (0)	-
Second trimester, n (%)	4 (18)	-
Third trimester, n (%)	18 (82)	-
**Toddler Characteristics**		
Age at evaluation, months	17 ± 2	17 ± 1
Male, n (%)	12 (55)	10 (59)

Race<br>White, n (%)<br>Black/African American, n (%)<br>More than one race, n (%)<br>Unknown or other, n (%)	 12 (55)<br>1 (5)<br>0 (0)<br>9 (41)	 11 (65)<br>0 (0)<br>3 (18)<br>3 (18)
Hispanic or Latinx, n (%)	16 (73)	6 (35)
Birth weight z-score[3]	-0.32 ± 0.86	0.27 ± 0.87
Birth length z-score[3]	0 ± 0.64	-0.10 ± 1.17
NICU admission, n (%)	5 (23)	2 (12)
Ever breastfed, n (%)	20 (91)	16 (94)

Values are expressed as mean ± standard deviation, median [interquartile range], or number of participants (percentage of group). [1]Perceived community social status was determined using the MacArthur Scale of Subjective Social Status, in which participants ranked their perceived standing in the community from 1 (lowest) to 10 (highest). Lower, middle, and upper tiers of perceived social status were conferred by scores of 1-3, 4-6, and 7-10, respectively. [2]Household food insecurity was determined by the U.S. Household Food Security Survey Module. [3]Z-scores were calculated using the Fenton growth chart, which accounts for gestational age and sex. *Abbreviations:* NICU, neonatal intensive care unit.
