# Supplementary material for: In Utero Exposure to Maternal SARS-CoV-2 Infection Is Associated With Higher Left Ventricular Mass in Toddlers
Source: Open Forum Infect Dis. 2024 May 31;11(6):ofae305. doi: 10.1093/ofid/ofae305 (PMC11204912; doi:10.1093/ofid/ofae305)
Supplement: ofae305_Supplementary_Data [file ofae305_supplementary_data.zip › Supplemental Table 2.pdf]

Supplemental Table 2. Association of In Utero SARS-CoV-2 Exposure with Left Ventricular Mass in Multivariable Models

	Left Ventricular Mass Index, $g/m^{2.7}$				Left Ventricular Mass/Body Surface Area, g/m^2			
	Basic Model		***Expanded Model***		***Basic Model***		***Expanded Model***	
**Parameter**	**ES (95% CI)**	***P*-value**	**ES (95% CI)**	***P*-value**	**ES (95% CI)**	***P*-value**	**ES (95% CI)**	***P*-value**
In utero SARS-CoV-2 exposure	10.5 (2.6, 18.4)	**0.01**	11.4 (2.9, 19.8)	**0.01**	10.4 (2.2, 18.5)	**0.01**	11.1 (2.4, 19.9)	**0.01**
Child age, months	-0.8 (-2.9, 1.3)	0.45	-0.9 (-3.0, 1.3)	0.43	-0.3 (-2.5, 1.9)	0.77	-0.4 (-2.6, 1.9)	0.73
Child male sex	3.8 (-3.5, 11.2)	0.29	3.6 (-4.0, 11.2)	0.34	4.3 (-3.3, 11.9)	0.26	4.0 (-3.9, 11.9)	0.31
Child Hispanic/Latinx ethnicity	-4.6 (-13.1, 4.0)	0.28	-3.4 (-12.8, 6.1)	0.47	-5.6 (-14.4, 3.3)	0.21	-4.3 (-14.1, 5.5)	0.38
Public insurance	5.3 (-5.2, 15.8)	0.31	5.7 (-5.4, 16.9)	0.30	5.7 (-5.2, 16.5)	0.30	5.7 (-5.8, 17.3)	0.32
Maternal college degree	3.8 (-6.8, 14.3)	0.47	4.1 (-7.4, 15.7)	0.47	3.2 (-7.7, 14.1)	0.55	3.0 (-9.0, 14.9)	0.62
Maternal pre-gravid obesity	-0.1 (-8.6, 8.3)	0.97	0.5 (-8.5, 9.4)	0.91	0.1 (-8.6, 8.8)	0.98	0.4 (-8.8, 9.6)	0.93
Breastfeeding	8.5 (-5.0, 22.1)	0.21	7.2 (-7.7, 22.1)	0.33	9.5 (-4.5, 23.6)	0.17	9.0 (-6.5, 24.4)	0.24
Preterm birth			-3.5 (-15.1, 8.2)	0.55			-2.0 (-14.1, 10.1)	0.74
Birth weight z-score[1]			1.4 (-3.4, 6.2)	0.55			1.7 (-3.2, 6.7)	0.48

Effect size (95% confidence interval) are shown for each covariate in basic and expanded models. Bold text denotes $P<0.05$ as the predefined threshold for statistical significance. [1]Z-score was calculated using the Fenton growth chart, which accounts for gestational age and sex. *Abbreviations:* CI, confidence interval; ES, effect size.
