# Supplementary material for: In Utero Exposure to Maternal SARS-CoV-2 Infection Is Associated With Higher Left Ventricular Mass in Toddlers
Source: Open Forum Infect Dis. 2024 May 31;11(6):ofae305. doi: 10.1093/ofid/ofae305 (PMC11204912; doi:10.1093/ofid/ofae305)
Supplement: ofae305_Supplementary_Data [file ofae305_supplementary_data.zip › Supplemental Figure 1.pdf]

Supplemental Figure 1: Participant Flow Diagram. Mothers who had previously enrolled in the Mass General Brigham (MGB) COVID-19 Pregnancy Biorepository during pregnancy were invited to participate in this pilot study with their toddlers (12-24 months). A total of 24 toddlers with in utero exposure to maternal SARS-CoV-2 infection and 19 toddlers whose mothers had no known history of SARS-CoV-2 infection and negative testing at delivery completed study screen to confirm eligibility. A total of 22 and 17 toddlers within each group, respectively, then underwent cardiometabolic assessment with data available for analysis. Figure was created with BioRender.com.
