# Supplementary figures and images for: In Utero Exposure to Maternal SARS-CoV-2 Infection Is Associated With Higher Left Ventricular Mass in Toddlers
Source: Open Forum Infect Dis. 2024 May 31;11(6):ofae305. doi: 10.1093/ofid/ofae305 (PMC11204912; doi:10.1093/ofid/ofae305)

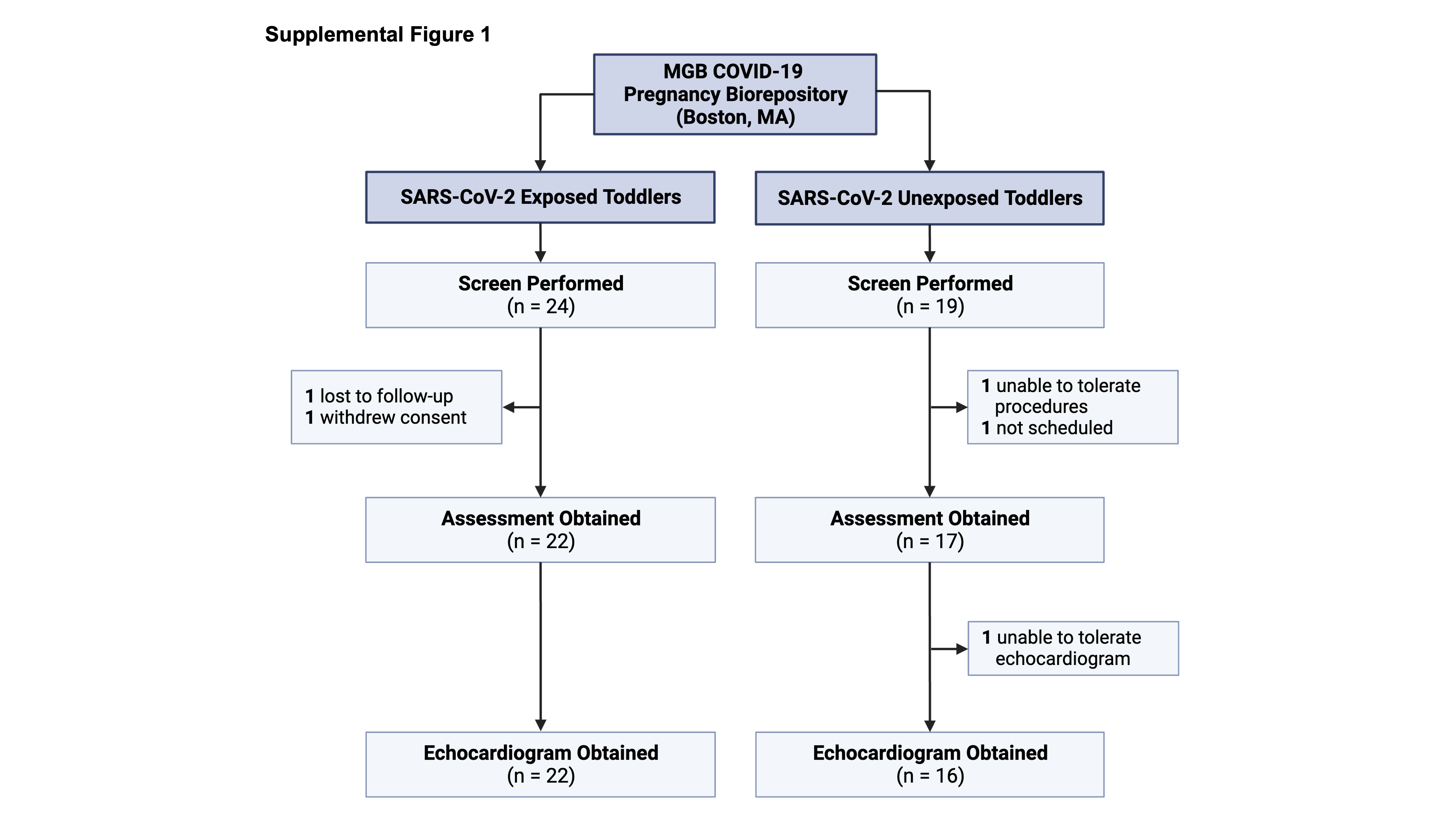

Supplement: ofae305_Supplementary_Data [file ofae305_supplementary_data.zip › Supplemental Figure 1.tiff]
